# Flower resource and land management drives hoverfly communities and bee abundance in seminatural and agricultural grasslands

**DOI:** 10.1002/ece3.3303

**Published:** 2017-09-05

**Authors:** Andrew Lucas, James C. Bull, Natasha de Vere, Penelope J. Neyland, Dan W. Forman

**Affiliations:** ^1^ Department of Biosciences Swansea University Swansea Wales UK; ^2^ National Botanic Garden of Wales Carmarthenshire Wales UK; ^3^ Institute of Biological, Environmental and Rural Sciences Aberystwyth University Aberystwyth UK

**Keywords:** bees, grassland, grazing, habitat management, hoverflies

## Abstract

Pollination is a key ecosystem service, and appropriate management, particularly in agricultural systems, is essential to maintain a diversity of pollinator guilds. However, management recommendations frequently focus on maintaining plant communities, with the assumption that associated invertebrate populations will be sustained. We tested whether plant community, flower resources, and soil moisture would influence hoverfly (Syrphidae) abundance and species richness in floristically‐rich seminatural and floristically impoverished agricultural grassland communities in Wales (U.K.) and compared these to two Hymenoptera genera, *Bombus,* and *Lasioglossum*. Interactions between environmental variables were tested using generalized linear modeling, and hoverfly community composition examined using canonical correspondence analysis. There was no difference in hoverfly abundance, species richness, or bee abundance, between grassland types. There was a positive association between hoverfly abundance, species richness, and flower abundance in unimproved grasslands. However, this was not evident in agriculturally improved grassland, possibly reflecting intrinsically low flower resource in these habitats, or the presence of plant species with low or relatively inaccessible nectar resources. There was no association between soil moisture content and hoverfly abundance or species richness. Hoverfly community composition was influenced by agricultural improvement and the amount of flower resource. Hoverfly species with semiaquatic larvae were associated with both seminatural and agricultural wet grasslands, possibly because of localized larval habitat. Despite the absence of differences in hoverfly abundance and species richness, distinct hoverfly communities are associated with marshy grasslands, agriculturally improved marshy grasslands, and unimproved dry grasslands, but not with improved dry grasslands. Grassland plant community cannot be used as a proxy for pollinator community. Management of grasslands should aim to maximize the pollinator feeding resource, as well as maintain plant communities. Retaining waterlogged ground may enhance the number of hoverflies with semiaquatic larvae.

## 1 INTRODUCTION

Pollination by insects is a key ecosystem service for both agriculture and natural systems (Gill et al., 2016; Klein et al., 2007; Vanbergen et al., 2013). However, there is considerable concern about the declines in pollination services worldwide, caused by agricultural intensification, habitat degradation, the spread of diseases and parasites, and climate change (Biesmeijer et al., 2006; Goulson, Nicholls, Botías, & Rotheray, 2015; Potts et al., 2010). In response to these concerns, a number of international, national, and regional initiatives have been proposed to address declines in pollinator populations (DEFRA 2014; IPBES 2016; Welsh Government 2013). A key part of these initiatives is the increasing understanding of the significant role of wild insect species in providing pollination services, both within agricultural and seminatural habitats (Garibaldi et al., 2013; Rader et al., 2015). There is an increasing body of research on how wild pollinators respond to agricultural management (Connelly, Poveda, & Loeb, 2015; Lüscher et al., 2016), and what management methods could be employed to restore pollinator populations (Bruppacher, Pellet, Arlettaz, & Humbert, 2016; Hardman et al., 2016; Kovács‐Hostyánszki et al., 2017). However, there are still gaps in our understanding of how pollinator populations respond to grassland communities and their management (Dicks et al., 2013; Mayer et al., 2011).

Among the Diptera, hoverflies (Syrphidae) are a significant group of pollinators (Rotheray & Gilbert, 2011). They can be effective pollinators of agricultural crops (Jauker & Wolters, 2008; Moisan‐Deserres, Girard, Chagnon, & Fournier, 2014; Prodorutti & Frilli, 2008) and wild plant species (Brown & McNeil, 2009; Forup, Henson, Craze, & Memmott, 2008; Ollerton, Winfree, & Tarrant, 2011). Individual hoverflies may not be as effective pollinators as bees, although this is compensated to some degree by population numbers (Jauker, Bondarenko, Becker, & Steffan‐Dewenter, 2012), and in some cases, the pollination service they provide can be complementary to that of bees (Ellis, Feltham, Park, Hanley, & Goulson, 2017). As adults they rely on nectar for carbohydrate, and pollen, which is a source of carbohydrate and lipids as well as protein for egg formation (Rotheray & Gilbert, 2011). There are 282 species of Syrphidae in Britain (Chandler, 2017), compared to 27 *Bombus* species and 247 other bee species (Falk & Lewington, 2015). Although hoverfly communities are known to be more species‐rich on organic farms (Power, Jackson, & Stout, 2016), relatively little is known of how hoverfly communities respond to some forms of agricultural intensification (Schweiger et al., 2007).

Seminatural grasslands are among the most threatened habitats in Europe, because they are readily subject to agricultural intensification, which substantially reduces plant diversity (Habel et al., 2013; Van Dijk, 1991) and their associated invertebrate communities (Hudewenz et al., 2012). In Great Britain, sites statutorily protected for their biodiversity (Sites of Special Scientific Interest) are selected and monitored primarily for their plant communities (Radcliffe, 1989), with the assumption that such habitats will deliver wider ecosystem services such as pollination (Eastwood et al., 2016; Garibaldi et al., 2011; Ricketts et al., 2008). Pollinator guilds might be expected to be more numerous in sites where the plant species diversity offers a more varied, abundant, and consistent food resource (Ebeling, Klein, & Tscharntke, 2011). Understanding how invertebrate populations, including hoverflies, respond to agricultural intensification in grasslands is essential in formulating strategies to support ecosystem services such as pollination in agricultural landscapes (Rzanny & Voigt, 2012; Weiner, Werner, Linsenmair, & Blüthgen, 2011).

The overarching aim of this study was to investigate which grassland habitats and management regimes might maximize hoverfly abundance and species richness and to compare this with the response of two bee genera, *Bombus* and *Lasioglossum*. As hoverflies have specific larval habitat requirements and feeding biology, they might be expected to respond differently than bees to grassland community. We used pan trapping to sample hoverfly and bee communities in grasslands in west Wales, UK, to answer the following questions:

1. How do hoverfly communities respond to both changes in grassland community as a consequence of agricultural intensification, and differences in plant community caused by variation in soil moisture. How does this response compare to two bee genera, *Bombus* and *Lasioglossum*? Since plant community richness has been shown to affect a number of invertebrate taxa (Schaffers, Raemakers, Sykora, & Ter Braak, 2008), we would predict that pollinator communities will be more abundant and species‐rich in grasslands with greater plant diversity.
2. How are hoverfly abundance and species‐richness influenced by flower resource and soil moisture, and do these factors operate in a similar manner with *Lasioglossum* and *Bombus*? Hoverflies have distinctive mouthparts compared to bees that influence which flower morphologies are accessible for feeding. We predicted that this would lead to differing responses to flower resource.
3. How are hoverfly communities in different grasslands structured, and how do environmental factors influence this? We predict that the diversity of hoverfly larval habitats and feeding biology would influence the species assemblages in different habitats.

## 2 MATERIALS AND METHODS

### 2.1 Site selection

**Figure 1:**
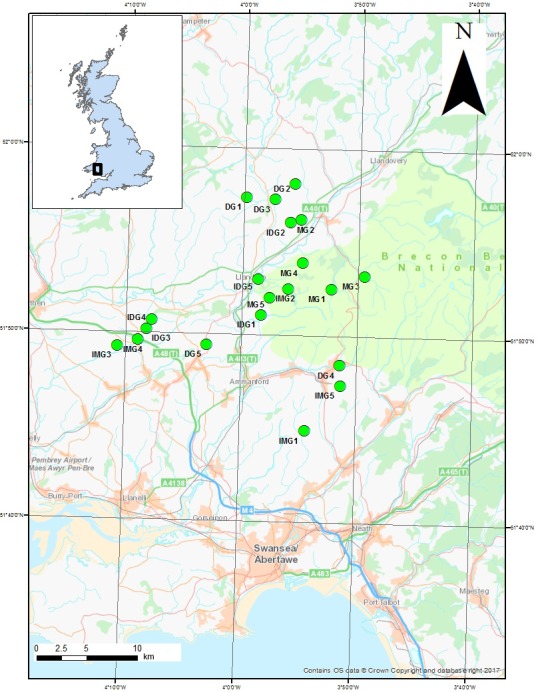
Sample site locations in South Wales, UK. (DG, dry grassland; IMG, improved dry grasslands; MG, marshy grasslands; IMG, improved marshy grasslands)

Site selection was based on National Vegetation Classification (NVC) community (Rodwell et al., 1991, 1992). Twenty grasslands in west Wales were selected for sampling between June 2011 and September 2011 (Fig. 1, see also Table S1). These consisted of five each of two conservation grasslands (NVC communities MG5 and M24) and two agricultural grasslands (NVC communities MG6 and MG10):

- MG5 *Cynosurus cristatus—Centaurea nigra* grassland (hereafter “dry grassland”, DG). A dicotyledon‐rich mesotrophic community frequently found in conservation grasslands in Britain, although rare in the wider agricultural landscape. Grasslands of this type are grazed or used for hay.
- M24 *Molinia caerulea—Cirsium dissectum* fen‐meadow (“marshy grassland”, MG). A species‐rich community found on moist peaty mineral soils in southern Britain. A relatively rare community, much reduced by agricultural improvement. Such grasslands are usually managed with grazing by cattle or horses.
- MG6 *Lolium perenne*—*Cynosurus cristatus* grassland (“improved dry grassland”, IDG). A grass‐dominated community that is the major permanent agricultural pasture in lowland Britain. These grasslands may be grazed by cattle, sheep or horses, or cut for silage/hay.
- MG10 *Juncus effusus—Holcus lanatus* rush—pasture (“improved marshy grassland”, IMG). A grass and rush dominated community developing on permanently moist agriculturally improved grasslands. Grasslands of this type are used for grazing cattle, sheep, or horses.

Sites ranged in size from 0.4 ha (site MG1) to 6.9 ha (site IDG3) (see Table S1), and were classified using existing survey information (Stevens & Mockridge, 2004), or by recording three standard NVC 2 × 2 m quadrats in order to assign the grasslands to an appropriate community (Rodwell et al., 1992). Plant species diversity for each grassland site derived from these samples is shown in Table 1. All of the grasslands studied were under grazing management, using combinations of cattle, horses, and sheep, but grazing was not under experimental control.

**Table 1:** Hoverfly abundance, hoverfly species richness, hoverfly Shannon H diversity, *Bombus* abundance, *Lasioglossum* abundance, site plant species richness, mean number of flowering species, mean flower score, and mean Ellenberg values for four grassland communities sampled using pan traps in 2011

Site name	Hoverfly abundance	Hoverfly species richness	Hoverfly Shannon *H*	Lasioglossum abundance	Bombus abundance	Site plant spp richness	Mean flowering species	Mean flower unit score	Mean Ellenberg *F*	Mean Ellenberg *R*	Mean Ellenberg *N*
DG1	212	12	1.821	31	69	29	9.5	217	5.2	5	3.3
DG2	7	5	1.550	1	17	23	2	16	5.4	5.6	4
DG3	15	9	2.026	4	34	27	3.5	35.5	4.9	5.1	4
DG4	6	4	1.523	0	9	20	5.5	21	4.3	5	3.5
DG5	25	11	1.804	11	17	30	9.5	125.5	4.8	4.9	3.3
IDG1	53	14	1.632	36	15	14	1.5	4.5	5.9	5.4	4.7
IDG2	9	7	1.831	24	7	15	2	4.5	5.6	5.5	4.6
IDG3	30	11	2.280	9	11	10	2.5	23	5.4	5.6	5
IDG4	25	6	1.167	5	17	9	2	14.5	5.6	5.2	4.7
IDG5	11	7	1.768	14	26	8	1.5	11	5.5	5.9	5.5
IMG1	59	15	2.220	4	21	18	5	77	6.1	5.6	4.2
IMG2	43	14	2.240	7	20	17	6.5	25.5	5.8	5.4	4.4
IMG3	50	14	2.401	6	9	12	3.5	96.5	6.3	5.5	5
IMG4	66	13	1.941	7	15	14	4.5	66.5	5.9	5.7	5.4
IMG5	18	8	1.769	1	12	11	5	197.5	6.8	5.4	4.5
MG1	64	11	1.247	3	16	27	4.5	25.5	6.4	4.2	2.6
MG2	37	11	1.986	6	29	30	4.5	91.5	5.4	3.6	2.3
MG3	192	19	2.189	31	58	21	8	334.5	6.7	3	1.6
MG4	179	22	2.166	8	34	24	8.5	118	6	3.4	2.1
MG5	48	15	2.237	0	14	21	5	119	7.2	3.7	2.1

### 2.2 Insect sampling

Insect sampling occurred in 2011 using pan traps. These consisted of plastic bowls (340 mm diameter and 128 mm depth), supplied by the manufacturer in three colors: white, blue, and yellow (Laubertie, Wratten, & Sedcole, 2006). Each sample site consisted of a group of nine pan traps at a single location, three of each color, on a metal stand that positioned the bottom of each trap at the height of the surrounding vegetation. They were surrounded by a three strand barbed wire fence to protect them from grazing animals, which would not impede insect movement (Wratten et al., 2003). Sample sites were at least 20 m from the nearest hedgerow to reduce the effect of hedgerow flowers attracting insects. The distance 20 m was selected because it was the maximum distance that a sample location could be placed from a hedgerow on the smallest sample site.

Sites were divided up into two equal groups to make fieldwork practical, with each group having a mix of grassland types. These two groups were sampled in alternate weeks (see Table S1). Each pan trap was filled with water to a depth of approximately 10 cm, to which approximately 0.25 ml of detergent and approximately 50 ml of ethylene glycol were added (Wheater & Cook, 2003). They were then left for 4 days and emptied within ±1 hr of the time they had originally been set. Insects were sieved from the water (sieve mesh size 2 mm^2^) and placed in bottles of 70% ethanol for identification. Pan traps were then covered or emptied for 10 days, before the next sample interval. We sampled each site six times at 14‐day intervals, between 17 June 2011 and 2 September 2011. Samples from each sample interval at a site were pooled for further analysis.

Insect samples were identified morphologically under a light microscope (×20–×40). Hoverflies were identified to species level using Stubbs and Falk (2002), bumblebees (*Bombus* spp) identified to species using Benton (2009), and honeybees *Apis mellifera* Linnaeus identified to species and solitary bees identified to genus using BWARS (2011).

### 2.3 Flower resource recording

The available flower resource (floral units) at each site was also measured. A 50 m × 50 m plot (or equivalent area, to allow for field shape) was set out in the center of each sample site. Thirty random sample locations were located within the plot. At each sample location, a 1 m × 1 m quadrat was placed, and the number of floral units of each forb species (herbaceous flowering plants, excluding grasses, sedges, and rushes) in the quadrat was recorded (Rose, 2006).

All flowers were counted on the plants within each sample location. For the Apiaceae, a single inflorescence was regarded as a floral unit. For *Narthecium ossifragum* L., *Rhinanthus minor* L., *Calluna vulgaris* L., and Orchidaceae species, a single flowering spike was regarded as a floral unit. Individual inflorescence heads of *Trifolium* species were also treated as a single floral unit. These measurements are similar to the “blossom units” of Dicks, Corbet, and Pywell (2002). Floral unit density measurements were recorded twice, between 1 June 2012 and 15 July 2012 and 16 July 2012 and 31 August 2012, and the mean of the count of floral units between the two sampling periods was calculated to give a “mean flower unit score” for each site. The mean number of plant species producing flowers between the two time period was also calculated to give a “mean flowering species” score (Table 1). For full details of plant species recorded flowering, see Table S2.

### 2.4 Environmental variables

The Ellenberg values for F (moisture), R (reaction or soil pH), and N (nitrogen) were collated for all grassland higher plant species recorded in NVC quadrats at each site (Hill, Mountford, Roy, & Bunce, 1999). The mean of these values was then calculated, to give a single value of F, R, and N for each site (Table 1). Altitude data for each site were obtained from 1:25,000 maps and site areas calculated using MapInfo ©Pitney Bowes Inc. Other environmental variables, such as rainfall or temperature, were not included as the sites were located relatively close together (Fig. 1).

### 2.5 Statistical analysis

Data from all pan traps were combined to give one result for each sample site, as the close proximity of the traps meant that the samples were not independent.

We calculated the number of hoverfly individuals (abundance), hoverfly species (species richness), and the hoverfly Shannon Diversity Score H for each site (Table 1). Hoverflies of the genus *Sphaerophoria,* which can only be identified to species in males, were grouped as one category “*Sphaerophoria* spp”. For the *Bombus* species, 430 individuals (99%) were identified to six common species (*B. hortorum* L.*, B. lapidarius* L.*, B. lucorum* L.*, B. pratorum* L.*, B. pascuorum* Scopoli, and *B. terrestris* L.). With so little species diversity, and the potential presence of the cryptic species *B. cryptarum* (Fabricius) and *B. magnus* (Vogt), all *Bombus* species were pooled each site, to give a single figure for *Bombus* abundance. For solitary bees, 69% (*n* = 299) of individuals belonged to one genus, *Lasioglossum*, with no other genus sufficiently widespread and numerous to justify further analysis. Honeybee abundance was not analyzed because of the possible bias in numbers that could be caused by any nearby domestic honeybee colonies.

As data for abundance, species richness, and diversity indices did not conform to a normal distribution, differences between the four grassland communities were assessed using Kruskal–Wallis H tests. This test was also used to investigate possible differences in altitude and site area between grassland types (see Table S3). All analysis was undertaken in IBM© SPSS© Statistics Version 22.

To investigate the influence of feeding resource availability on hoverfly abundance and species richness, generalized linear modeling using a Poisson distribution and a log link function was undertaken. We accounted for overdispersion by employing a quasipoisson model where appropriate. Poisson models were assessed using chi‐squared tests, quasipoisson using *F* tests. The response variable comprised count data (abundance or richness), with natural logarithm‐transformed floral unit scores (transformed as maximum floral unit scores were substantially lower in agriculturally improved land), soil moisture (marshy vs. dry), and level of improvement (unimproved vs. improved) as explanatory variables, which were included as main effects as well as fully interacting. Analysis was undertaken using R 3.1.4 (R Core Team 2014).

Hoverfly community structure was visualized using canonical correspondence analysis (CCA), using Environmental Community Analysis Version 2.1, 2007, Pisces Conservation Ltd. Lymington, UK (www.pisces-conservation.com). Mean site Ellenberg values for plant species from each sample site were used as explanatory variables in the model (Cajo, 1986), together with mean floral unit score and mean flowering species. Weighted variables were used to generate the ordination figures. A Monte Carlo randomization test, using 1,000 trials, was undertaken to test the significance of the variability explained by each ordination axis.

## 3 RESULTS

In total, 1,171 hoverflies of 42 species, 450 *Bombus* bees of 10 species, and 299 solitary bees in 12 genera were recorded (Tables 1 and S4).

Among hoverflies, *Eristalis* species were the most frequent (45% *n* = 520) across all sites, with *Helophilus pendulus* L. (24% *n* = 320) and *Rhingia campestris* Meigen (9% *n* = 106) also commonly recorded.

There were no significant differences in altitude or site area between grassland types using a Kruskal–Wallis test, and therefore, these factors were not used in subsequent modeling (Altitude: H_(3)_ = 6.56 *p* > .05, Area H_(3)_ = 2.78 *p* > .05).

**Figure 2:**
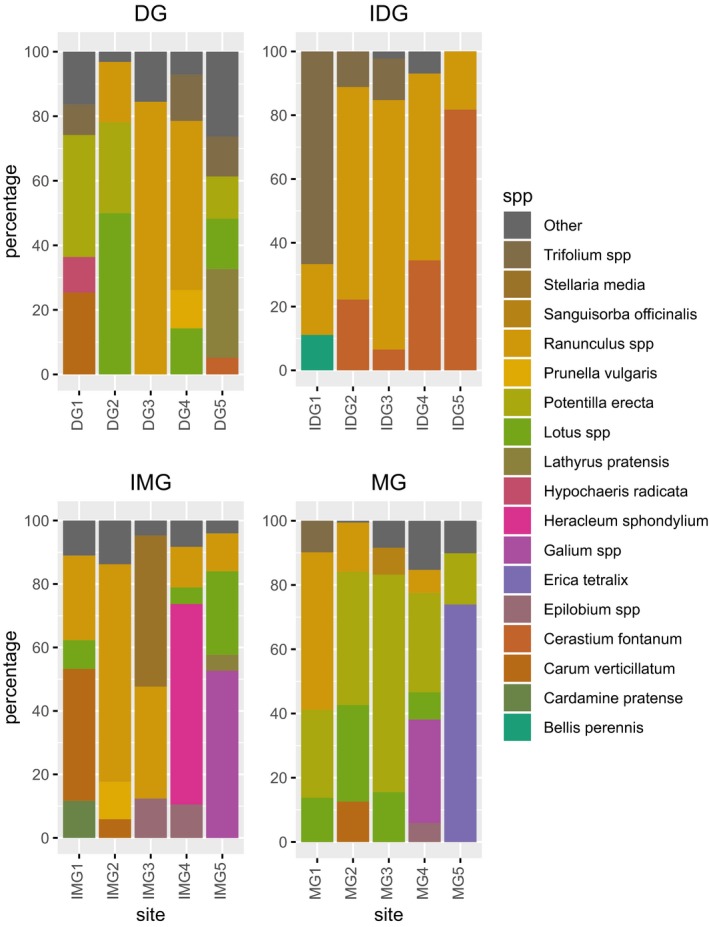
Percentage of flower units from plant species contributing more than 5% of total flower unit score in four grassland communities (DG, dry grassland; IDG, improved dry grassland; IMG, improved marshy grassland; MG, marshy grassland

A summary of the floral unit scores is shown in Fig. 2 and Table 1 (with full results in Table S2). A total of 45 species were recorded flowering across all sites. Among the most widespread flowering taxa were *Ranunculus* spp, *Potentilla erecta*, and species of Apiaceae (*Heracleum sphondylium* and *Carum verticillatum*).

### 3.1 Are there differences in hoverfly diversity and abundance, and bee abundance, between grassland types?

No significant differences were observed in hoverfly abundance, H Diversity, *Bombus* abundance, and *Lasioglossum* abundance between grassland community types, using a Kruskal–Wallis test (see Table S3). There was an initial significant difference in hoverfly species richness between grassland communities (*H* = 8.225, *p* = .042). However, subsequent analysis using a Bonferroni correction for multiple comparisons showed no significant pairwise comparisons (see Table S3).

### 3.2 Are hoverfly abundance and species richness, and bee abundance, influenced by flower resource and soil moisture?

#### 3.2.1 Treatment of “mean floral unit score” and “mean flowering species”

We compared “mean floral unit score” and “mean flowering species” as measures of flower resource. Both variables were positively correlated (Spearman's ρ = 0.797, *p* < .001). Therefore, we did not include both as explanatory variables in the same statistical models. Instead, we compared the full model (three‐way interaction: flower score × improvement × moisture) for each measure of flowering, using AIC. We found that for all insect pollinator taxa, “mean floral unit score” was a better predictor than “mean flower species” (Hoverfly abundance, ΔAIC = 7.24. Hoverfly species richness ΔAIC = 9.27. *Lasioglossum* abundance, ΔAIC = 10.15. *Bombus* abundance, ΔAIC = 2.83).

#### 3.2.2 Insect pollinator abundance and species richness

**Figure 3:**
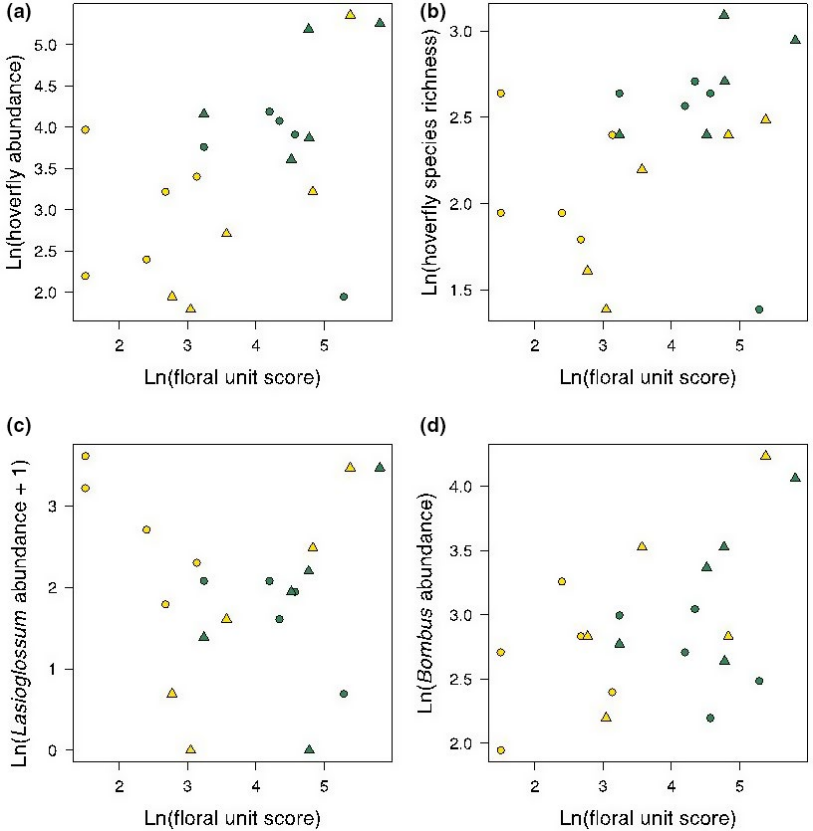
Insect abundance and species richness assessed by pan trapping in four grassland habitats, between June and August 2011. Data points represent natural logarithm‐transformed aggregate abundances from nine pan traps, with three replicates of each color (blue, white, yellow) pan colors. Panel a—hoverfly abundance. Panel b—hoverfly species richness. Panel c*—Lasioglossum* abundance. Panel d*—Bombus* abundance. Marshy grasslands—green triangles; Improved marshy grasslands—green circles; Dry grasslands—yellow triangles; Improved dry grasslands—yellow circles

The effects of mean floral unit score on hoverfly abundance, hoverfly species richness, *Lasioglossum* bee abundance, and *Bombus* bee abundance, were quantified (Fig. 3). In each case, the full model incorporating the three‐way interaction between floral unit score, agricultural improvement, and soil moisture was assessed by stepwise deletion using *F* tests (Table 2). In all cases, the best fitting model showed a statistically significant increase in pollinators with increasing mean floral unit score in unimproved grassland (Table 3). However, this was not found in agriculturally improved grassland (Table 3). Hoverfly abundance, hoverfly species richness, and *Bombus* bee abundance were not significantly affected by mean floral unit score, whereas *Lasioglossum* bee species abundance significantly decreased with increasing mean floral unit score (Table 3). Other interaction terms were not statistically significant. As it was not a component of statistically significant interaction terms, soil moisture was assessed as a main effect (Table 2). This was only found to have a statistically significant effect on hoverfly species richness, with more species found in marshy ground than dry (Estimate = 0.542, *SE* = 0.181, *F*_1,15_ = 2.99, *p* = .009).

**Table 2:** Analysis of Variance explaining insect pollinator abundance and species richness. FS, mean Floral unit Score; I, agricultural Improvement; and M, soil moisture. Terms are presented in the order they were assessed in stepwise deletion of the full model, incorporating the three‐way interaction and all lower order terms. Statistically significant terms (*p* < .05) are shown in bold

	Hoverfly abundance		Hoverfly species richness	
	*F*	*p*	*F*	*p*
FS × I × M	0.761	.400	0.095	.763
I × M	0.292	.598	0.047	.832
FS × M	4.124	.062	1.495	.242
M	0.815	.381	**9.277**	**.008**
FS × I	**5.960**	**.027**	**10.75**	**.005**

	*Lasioglossum* abundance		*Bombus* abundance	
	*F*	*p*	*F*	*p*
FS × I × M	0.569	.465	0.595	.455
I × M	2.185	.163	0.169	.687
FS × M	0.691	.420	0.330	.575
M	1.821	.197	0.900	.358
FS × I	**76.84**	**<.001**	**6.868**	**.019**

**Table 3:** Parameter estimates from log‐linear regression of insect pollinator abundance and species richness on mean floral unit score, in agriculturally improved and unimproved grassland. Statistically significant terms are shown in bold

	Estimate	*SE*	*t*	*p*
Response to floral unit score in agriculturally improved land				
Hoverfly abundance	0.111	0.225	0.494	.628
Hoverfly species richness	−0.160	0.102	1.571	.137
*Lasioglossum* abundance	−**0.686**	**0.133**	**5.162**	**<.001**
*Bombus* abundance	−0.016	0.136	0.118	.908
Response to floral score in agriculturally unimproved land				
Hoverfly abundance	**0.901**	**0.240**	**3.753**	**.002**
Hoverfly species richness	**0.274**	**0.105**	**2.601**	**.020**
*Lasioglossum* abundance	**1.253**	**0.223**	**5.612**	**<.001**
*Bombus* abundance	**0.480**	**0.134**	**3.585**	**.002**

### 3.3 Hoverfly community structure

There was a substantial degree of multicollinearity between R (reaction) and N (nitrogen) (Reaction: *R* ^2^ = 0.906, VIF = 10.65; Nitrogen: *R* ^2^ = 0.900 VIF = 9.96). As this study was concerned with the impact of agricultural improvement, the variable N was retained in the analysis and R was removed.

A Monte Carlo significance test with 1,000 runs showed that axis 1 (broadly defined by nitrogen, N and mean number of flowering species) was significant in explaining the variance of the data, while axis 2 was not significant (Axis 1 Eigen values = 0.269, mean = 0.168, maximum = 0.303, minimum = 0.076, *p* = .015; Axis 2 Eigen values = 0.093, mean = 0.095, maximum = 0.162, minimum = 0.047, *p* = .510).

**Figure 4:**
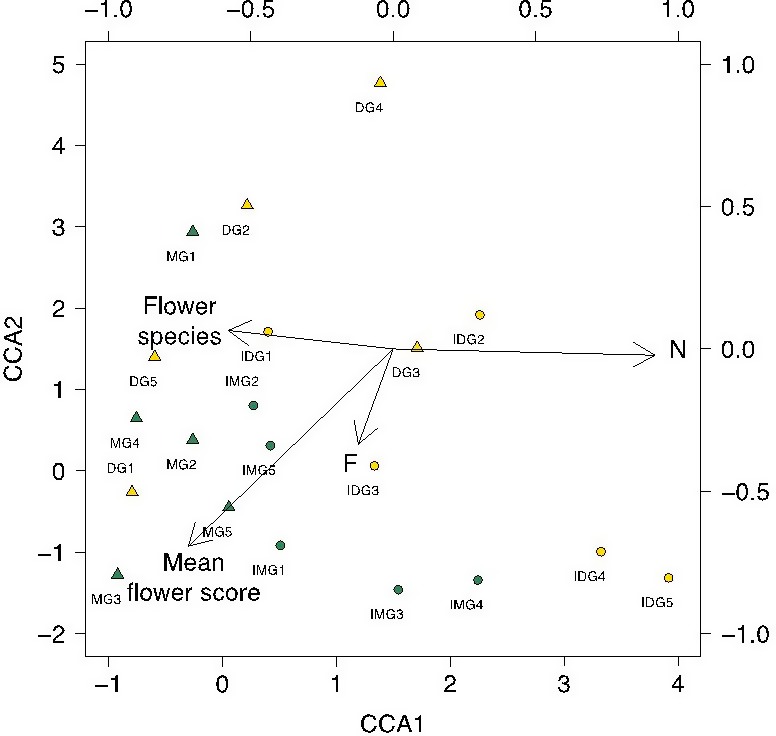
Canonical correspondence analysis biplot of hoverfly communities assessed using pan traps during 2011 at four grassland habitats, using mean flower score, mean number of flowering species (“Flower species”), and mean plant Ellenberg values for F (moisture) and N (nitrogen) as environmental variables. MG (marshy grasslands)—green triangles; IMG (improved marshy grasslands)—green circles; DG (dry grasslands)—yellow triangles; IDG (improved dry grasslands)—yellow circles

The marshy grassland and improved marshy grassland showed within‐group clustering on axis 1, suggesting a consistent community of hoverflies (Fig. 4). The dry grassland sites were also clustered on axis 1. The improved dry grassland hoverfly communities showed relatively low clustering on axis 1, suggesting there is no consistent hoverfly assemblage associated with this habitat.

**Figure 5:**
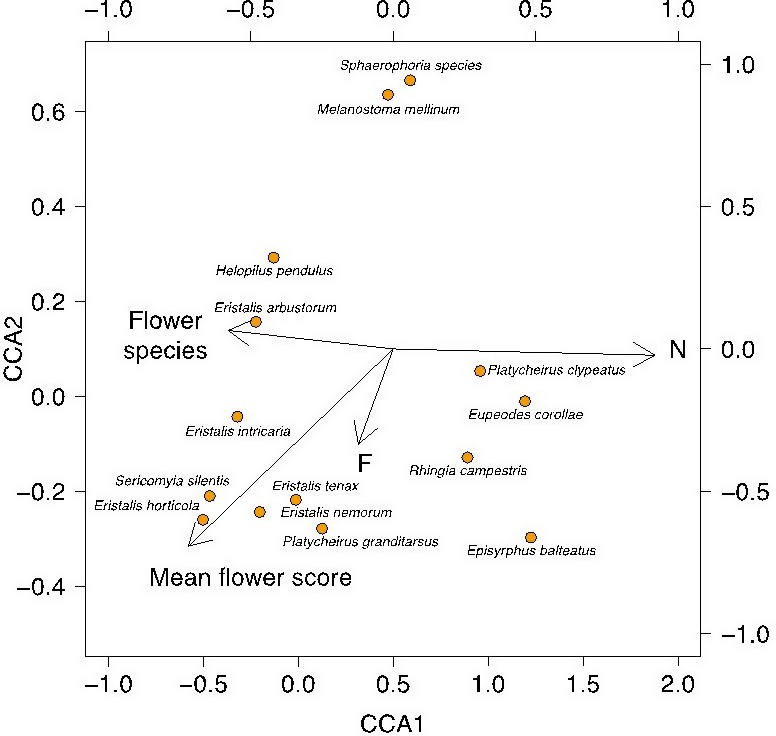
Canonical correspondence analysis biplot of hoverfly species assessed using pan traps during 2011 at four grassland habitats, using mean flower score, mean number of flowering species (“Flower species”), and mean plant Ellenberg values for F (moisture) and N (nitrogen) as environmental variables. For clarity, species with an abundance less than 1% of the total for all sites have been omitted

Common hoverfly species with semiaquatic larvae (*Eristalis* species, *Helophilus pendulus,* and *Sericomyia silentis* Harris) or species commonly occurring in wet pastures (*Platycheirus granditarsus* Forster) were associated with marshy grassland (Fig. 5), having low values on axis 1. By contrast, *Rhingia campestris*, whose larvae use cow dung, and *Episyrphus balteatus* De Greer, *Eupeodes corollae* Fabricius, *Sphaerophoria* species, *Melanostoma mellinum* L., and *Platycheirus clypeatus* Meigen, all of which have aphidophagous larvae, have higher values on axis 1, suggesting a higher association with improved pastures.

## 4 DISCUSSION

This study shows that hoverflies and bees are responding to both the habitat and its flower resource. Our prediction was that the grassland community, as defined by plant species richness, would have a key influence on the abundance and species richness of their hoverfly communities. However, it is the flower abundance, as well as the soil moisture associated with different grassland communities, which is critical to determining the abundance and species composition of hoverfly communities.

### 4.1 Hoverflies, bees, and grassland community type

There were no significant differences in hoverfly abundance, species richness, or bee abundance between the four grassland communities (“dry grassland” DG, “improved dry grassland” IDG, “improved marshy grassland” IMG and “marshy grassland” MG). This was unexpected, given the evidence that diverse plant communities support equally diverse invertebrate communities (Schaffers et al., 2008; Senapathi et al., 2015). Diverse grasslands offer more consistent foraging resources to all pollinator guilds, which can enhance the stability of pollination services (Garibaldi et al., 2011; Ockinger & Smith, 2007). However, the results of this study suggest that plant community alone cannot be used as a surrogate for the associated pollinator assemblage.

### 4.2 Hoverflies, flower resource, and soil moisture

Increasing the flower resource, and therefore the feeding opportunities for adult hoverflies, increased both hoverfly abundance and species richness. This effect was only detected on unimproved grasslands, and the absence of this effect on improved grasslands may be a result of three factors. Firstly, improved grasslands, by definition, have a lower forb and higher graminoid cover (Rodwell et al., 1992), and therefore will have low flower scores overall. Secondly, the plant species that were flowering at improved sites included species such as *Cerastium fontanum*,* Stellaria media*, and *Galium* species (e.g., sites IMG3, IMG5, and IDG5, Figs 1 and 2). These species produce a large number of very small flowers, with relatively low nectar production (Baude et al., 2016). Such nectar splitting can make food collection more energetically demanding (Carvalheiro et al., 2014). Thirdly, other species that form a significant part of the limited flower resource at improved sites include *Trifolium* species and two genera of Fabaceae, *Lathyrus*, and *Lotus* (e.g., IDG1, IMG3, and IMG5, Figs 1 and 2). These plants produce zygomorphic flowers that are relatively inaccessible to the mouthparts of many hoverfly species (Branquart & Hemptinn, 2000; Gilbert, 1981). Thus, not only is the feeding resource for hoverflies reduced on improved grasslands, but many of the flowers that are present are of low quality as a food resource. A similar effect of increasing flower resource was also found for *Lasioglossum* and *Bombus* bees, which were both more abundant at sites with higher flower scores. However, abundance of *Lasioglossum* fell with increasing flower score on improved dry grasslands. This could represent competitive interaction by other pollinators (Biesmeijer, Richter, Smeets, & Sommeijer, 1999; Dworschak & Bluthgen, 2010), the flower species and available nectar resource (as described for hoverflies above), or differences in foraging strategies (Gathmann & Tscharntke, 2002).

Flower resource is dependent upon both the availability of suitable plant species and a suitable management. Intensive management can result in a more uniform sward with few flowers (Vickery et al., 2001). This can make a species‐rich grassland little different, in terms of the available flowers for pollinator foraging, from an agriculturally improved grassland (Power & Stout, 2011). This may explain the lack of significant differences in hoverfly abundance and species richness, and bee abundance, between the different grassland communities. The plant species composition of a grassland community itself is not a reliable predictor of pollinator abundance and species richness unless the management regime and consequent flower resource are also considered (Feltham, Park, Minderman, & Goulson, 2015; Jönsson et al., 2015; Power & Stout, 2011). Soil moisture level did not influence hoverfly abundance, species richness, or bee abundance.

### 4.3 Hoverfly community structure and grassland type

The results of the CCA showed that axis 1, broadly associated with environmental variable N and mean number of flowering species which are both a proxy for the degree of agricultural improvement (Habel et al., 2013), is a key factor in determining hoverfly community structure, demonstrating the importance of retaining agriculturally unimproved pastures as hoverfly habitat.

There appears to be a consistent community of hoverflies associated with both marshy grasslands and, to a lesser extent, improved marshy grasslands. Dry grassland sites also show some degree of clustering on axis 1 (Fig. 4). Hoverflies with semiaquatic larvae (*Eristalis* species, *Helophilus pendulus*, and *Sericomyia silentis*) or species associated with damp pastures (*Platycheirus granditarsus*) (Stubbs & Falk, 2002) were particularly associated with marshy and improved marshy grasslands (Fig. 5). This indicates that these grasslands may provide species which oviposit in waterlogged sites with localized egg‐laying sites and suitable larval habitat, not reflected in the wider plant community. In contrast, the hoverfly assemblages in dry grasslands were more variable in species composition compared to marshy grasslands and included species with carnivorous larval stages (*Melanostoma mellinum*,* Eupeodes corollae* Fabricius*,* and *Episyrphus balteatus* De Geer). It is also noticeable that *Rhingia campestris*, a species whose larval habitat is cow dung, has a greater tolerance for relatively higher *N* values than many other species. This may reflect cattle husbandry in a range of grasslands, and the ability of *R. campestris* adults to feed on flowers inaccessible to many other hoverflies (Haslett, 1989). Larval habitat has previously been noted as a factor structuring hoverfly community structure (Meyer, Jauker, & Steffan‐Dewenter, 2009; Mueller & Dauber, 2016). Given the diverse nature of hoverfly larval strategies and their potential agricultural importance, greater study of larval ecology is a subject for future research.

Improved dry grassland hoverfly communities showed relatively low clustering in their species assemblages on axis 1 (Fig. 4). The hoverfly species present appear to be a stochastic association of species, with no clear or repeatable pattern between sites. If hoverflies from more suitable habitats were dispersing into improved dry grasslands, it might be expected that the hoverfly community composition of improved dry grasslands might reflect more species‐rich hoverfly communities, although probably at lower levels of abundance. That the hoverfly communities of these floristically impoverished habitats vary among each other, and have an unpredictable element, suggests that there is no consistent “spill‐over” into improved dry grasslands from more suitable, if distant, habitats. This indicates that, although the delivery of pollination services by hoverflies in agriculturally intensive systems is related to the amount of available habitat in the wider landscape (Power et al., 2016), it cannot rely on dispersal from distant breeding sites.

Measuring floral unit abundance is relatively straightforward for land managers but, as shown by this study, has limitations if the accessibility of the nectar resource is not considered. Using nectar resource directly would be a more robust method, particularly as data on many common British agricultural species are now available (Baude et al., 2016). Unfortunately, the data does not include the umbellifer *Carum verticillatum*, a common plant in our study found in seminatural and even some improved marshy grasslands in west Wales. However, integrating flower abundance and nectar resource is likely to improve the ability of models to predict hoverfly communities compared to flower unit data. Similarly, using pan traps is a simple and effective method of sampling pollinator populations (Carvell et al., 2016), but does have limitations. Unlike netting insects as they visit plants, there is no direct link between pan trap records and flower visitation (Popic, Davila, & Wardle, 2013). Pan traps can also oversample pollinators in resource‐poor environments by “sucking in” pollinators and can undersample in flower‐rich sites where there are many competing stimuli (Hickman, Wratten, Jepson, & Frampton, 2001; Roulston, Smith, & Brewster, 2007; Wilson, Griswold, & Messinger, 2008). However, they do reduce the sampling bias associated with hand netting (Spafford & Lortie, 2013). Ideally, any site pollinator assessment should use a combination of trapping and net sweeping to collect data.

This study attempted to control for the influence of the landscape on pollinator populations by selecting sites that were relatively distant from other habitats from which pollinators might disperse. However, no such control can be perfect, and wider landscape has been demonstrated to have an impact on pollinator populations at specific sites (Ekroos, Rundlof, & Smith, 2013; Ockinger, Lindborg, Sjodin, & Bommarco, 2012; Power et al., 2016). Therefore, the possibility that some of the differences in hoverfly communities in this study were the result of factors operating at a landscape scale cannot be discounted.

### 4.4 Grassland hoverfly community assessment

This study provides a framework to assess the potential for a grassland to support a diverse hoverfly community. Grassland plant community has been long treated as a surrogate for invertebrate community richness, for example, with ground beetles (Yanahan & Taylor, 2014), and butterflies, and grasshoppers (Koch et al., 2013). Plant communities have been frequently used as a method of selecting sites for nature conservation designations, both at a British and at European levels (Evans, 2012; Mucina et al., 2016; Radcliffe, 1989). This study suggests that a more diverse plant community has the potential to support a rich hoverfly fauna, but only if management meets other key requirements of their lifecycle, such as flower resource.

Flower resource is a function of both the plant community and the associated management regime. While agricultural improvement can reduce the number of forb species directly, any factor that can reduce the numbers of flowers, even on floristically species‐rich swards, can have a direct effect on flower resource availability, and therefore hoverfly abundance and species richness. Grazing is one such a factor, and a response to grazing has been noted in a number of other invertebrate groups, including dung beetles, (Verdu et al., 2007), butterflies, and grasshoppers (Jerrentrup, Wrage‐Monnig, Rover, & Isselstein, 2014). Similar moderate grazing regimes have been shown to be beneficial for pollinator communities (Vanbergen et al., 2014) and specifically hoverflies (Hudewenz et al., 2012; Lazaro, Tscheulin, Devalez, Nakas, & Petanidou, 2016). This study confirms that a resource of flowers available for feeding hoverflies, and the lower intensity management regime that can help produce it, is a significant factor in driving hoverfly communities.

Dicks et al. (2015) attempted to evaluate how much suitable habitat is required to maintain viable populations of wild bees, in order to maintain a viable pollination ecosystem service. Our findings suggest that a similar calculation for hoverflies would have to take some account of larval habitat requirements, an effect that has been noted in relation to other insect providers of ecosystem services, such as parasitoid wasps (Gillespie, Gurr, & Wratten, 2016). Hoverfly communities of marshy grasslands, whether agriculturally improved or not, can be distinctive from those found in drier grasslands (Carey, Williams, & Gormally, 2017). In particular, our study has shown that hoverflies in the genera *Eristalis*,* Sericomyia*, and *Helophilus*, all appear to be particularly associated with wetter ground. As these are relatively large, hairy bee and wasp mimics (Stubbs & Falk, 2002), they may have significant potential as pollinators (Stavert et al., 2016).

Habitats that support hoverfly populations provide a pollination ecosystem service, by providing pollinators to crops on adjacent land (Garibaldi et al., 2011), facilitating additional functions that underpin other ecosystem services (Mace, Norris, & Fitter, 2012), and maintaining the cultural services provided by the habitats themselves (Potts et al., 2016). To effectively conserve and enhance the pollination ecosystem service provided by hoverflies, management should retain remaining species‐rich grassland communities (Lentini, Martin, Gibbons, Fischer, & Cunningham, 2012; Ockinger & Smith, 2007) and ensure they are under appropriate management that allows a sufficient flower resource for feeding. Grasslands that may have been subject to agricultural improvement can still be of some value to hoverflies if management becomes less intensive, allowing more forbs with accessible food resources to flower (Hudewenz et al., 2012; Orford, Murray, Vaughan, & Memmott, 2016). Finally, and critically, management for varied hoverfly communities must include the provision of larval habitat. For semiaquatic species, this can include either land that is periodically waterlogged or adjacent wetlands.

## AUTHOR CONTRIBUTIONS

Andrew Lucas conceived the study, undertook fieldwork, all data collection, statistical analysis, and drafted the manuscript. James C Bull undertook statistical analysis and drafting of manuscript. Natasha de Vere advised on drafting the manuscript. Penelope J Neyland advised on statistical analysis and drafting the manuscript. Dan W Forman helped to conceive the study, advised on statistical analysis, and helped draft the manuscript.

## CONFLICT OF INTEREST

None declared.

## DATA ACCESSIBILITY

Data are available via the Dryad Digital Repository https://doi.org/10.5061/dryad.1rd15.
